# Evaluation of clinical impact of Interleukin 8 gene expression in patients with fibromyalgia

**DOI:** 10.1186/s42358-025-00453-8

**Published:** 2025-04-23

**Authors:** Carolina Dias da Silva Amorim, Marco Felipe Macêdo Alves, Marcella Dias Teixeira, Larissa Vasconcelos Queiroz, Thaysa Walleria de Aragão Santos, Aline Ranzolin, Danyelly Bruneska Gondin Martins, Claudia Diniz Lopes Marques

**Affiliations:** 1 Programa de pós-graduação em Saúde Translacional, Centro de Ciências Médicas, Universidade Federal de Pernambuco, Recife-PE, CEP 50760-901 Brazil; 2 Programa de residência médica em reumatologia do Hospital das Clínicas/Ebserh, Universidade Federal de Pernambuco, Recife-PE, CEP 50760-901 Brazil; 3 Instituto Keizo Asami (iLIKA), Centro de Biociências, Universidade Federal de Pernambuco, Recife, CEP 50760-901 Brazil; 4 Hospital das Clínicas, Universidade Federal de Pernambuco, Empresa Brasileira de Serviços Hospitalares (Ebserh), Recife-PE, CEP 50760-901 Brazil

**Keywords:** Fibromyalgia, Interleukin 8, Depression, Fatigue, Sleep quality, Obesity

## Abstract

**Background:**

Fibromyalgia (FM) is a musculoskeletal syndrome characterized by diffuse and chronic pain associated with other symptoms such as fatigue, sleep/cognition disorders, headache, depression and anxiety, resulting from a change in pain processing. Previous research has shown an increase in some interleukins (IL) in patients with FM when compared to controls, however, there is still no uniformity and consensus in the results. There is no study that evaluates *IL8* mRNA expression in FM and its association with obesity and other clinical parameters. This study aims to verify the impact of *IL8* mRNA expression on the clinical parameters of patients with FM (FMG) in relation to the comparison group (CG).

**Method:**

This study evaluated patients diagnosed with FM treated at the rheumatology service of the Hospital das Clínicas of the Federal University of Pernambuco (HC-UFPE). The CG group was composed of individuals without chronic pain (companions of the patients and hospital employees). Clinical and demographic data were collected in both groups, and questionnaires for fatigue (FACIT-F), impact of FM (FIQ-R), depression (BDI), and sleep (NRS) were applied to both groups. Peripheral blood was collected for evaluation of *IL8* gene expression through real time polymerase chain reaction (qPCR).

**Results:**

Patients with FM show a lower frequency of *IL8* gene expression compared to the CG, but FMG presented mainly up regulated in relation to CG. There was no association of *IL8* expression and worse FIQ-R indices, sleep disturbance, BMI or fatigue. However, there was an association between *IL8* expression and moderate depression (*p* = 0.002) and physical activity (*p* = 0.039), where patients in FMG who did express *IL8* were practicing less physical activity.

**Conclusion:**

Patients in FMG did not have a higher frequency of *IL8* expression compared to CG, however patients with *IL8* expression have a greater association with moderate depression.

## Introduction

Fibromyalgia (FM) is a chronic non-inflammatory pain syndrome characterized by diffuse pain [1] associated with other symptoms such as fatigue, mood and sleep disorders, headache and cognitive impairment, among others [2]. Although some factors are known to predispose an individual to develop FM, such as physical trauma, emotional events, and genetic factors, its exact etiology is still unknown [2, 3]. To be diagnosed with FM, the patient must present with diffuse pain for more than 3 months and associated symptoms (fatigue, sleep disturbances, cognitive alterations, headache, depression, or abdominal pain) of significant intensity, scoring a specific number in a questionnaire validated for the disease [4]. Among the criteria that define the pathology, there is no characteristic laboratory or imaging data, and the diagnosis is made only with the clinical history and physical examination [2].

Although inflammation and cell destruction are correlated with the pain response, chronic pain is probably not the direct consequence of acute inflammation or cell damage [5, 6]. One of the main hypotheses investigated in FM refers to changes in nociceptive mechanisms, related to pain modulation, which would result in hyperalgesia and/or allodynia [7]. Some works have evaluated the hypothesis of participation of immunological mechanisms in the genesis and evolution of pain in FM, but the results found are also contradictory and so far, do not allow confirming or excluding this hypothesis [8]. So far, alteration of several markers like cytokines has been demonstrated, but without consensus in research. Some of the interleukins (IL) studied in FM and chronic pain are: IL1β, IL6, IL8, IL10 and IL15 [9, 10]. More recently, an association of chronic pain with high levels of IL8 has been found, but studies are still not conclusive [11]. In 2019, work was carried out with murine models using the drug Reparixin, an IL8 antagonist, which was able to reduce chronic low back pain [12].

In addition to the relationship with chronic pain, these inflammatory mediators are involved in other pathologies, and it has already been documented that there is an increase in some interleukins such as IL6 and IL8 in obesity [13]. Among prognostic factors of FM, obesity is the most prevalent comorbidity in FM patients compared to healthy population [14]. In addition to prevalence, some studies have shown a relationship between obesity and worse FM indices through the FIQ-R [15]. Several studies have already demonstrated an increase in c-reactive protein (CRP) and other cytokines such as IL8 in patients with obesity, however, only in 2012 this relationship of inflammatory biomarkers and obesity was seen in patients with FM [16]. Since then, other studies have tried to evaluate serum cytokines levels and its association with FM symptoms, but there is no data regarding *IL8* gene expression, only serum levels. Considering that cytokines levels are a challenge to be detected because of dynamic secretion processes and short half-lives [9], our group aimed to examine if the *IL8* gene expression is considered to have less changes among circumstances. There is no data evaluating *IL8* gene expression in FM and its possible association with obesity and clinical manifestations such as depression, sleep disturbance and fatigue.

## Methodology

### Study design

A cross-sectional, observational, analytical study was carried out from August to December of 2021, at the FM outpatient clinic at the rheumatology service of Hospital das Clínicas of the Federal University of Pernambuco (HC-UFPE). The sample was formed by convenience, according to the number of patients treated at the service and cost with material for the evaluation of gene expression. The case group (FMG) was selected from patients undergoing regular clinical follow-up at the specialized FM outpatient clinic, according to the inclusion/exclusion criteria. The comparison group (CG) was composed of individuals without chronic pain and who did not use anti-inflammatory or corticosteroids and were selected from hospital employees and patients’ companions.

Research variables were the result of IL8 gene expression in FMG and CG, BMI calculation, in addition to the values ​​obtained from different questionnaires for: fatigue (FACIT-F), impact of FM (FIQ-R), depression (BDI), and sleep (NRS). The dependent and independent variables were not defined because there is no definition in the literature regarding IL8 gene expression in the healthy population and in patients with chronic painful disease. The normal values ​​have not been established, as well as the relationship between gene expression and serum values.

The FMG were clinically evaluated and answered the study protocol questionnaires: BDI, FIQ-R, FACIT-F and NRS. In the CG, the following questionnaires were applied: BDI, FACIT-F and NRS.

### Inclusion and exclusion criteria

In FMG, the study included women diagnosed with fibromyalgia by 2016 criteria from American College of Rheumatology, being more than 18 years old. For CG was included women with more than 18 years old who do not have chronic pain matched by age. It was excluded participants with overlapping syndromes, current infection, undergoing pregnancy or who had taken anti-inflammatory or corticoid in the last 7 days.

### Collection instruments

The Functional Assessment of Chronic Illness Therapy (FACIT-F) is an instrument to assess fatigue, initially developed for cancer patients and later adapted for several chronic diseases, such as Lupus, Rheumatoid Arthritis and Anemia. It consists of 13 questions that assess fatigue through the physical and psychological condition of the patient. The questionnaire ranges from 0 to 52 and the higher the result, indicates a lower level of fatigue. It was validated and adapted to the Portuguese language in 2015 by Zumpano [17].

Fibromyalgia Impact Questionnaire Revised (FIQ-R) is a questionnaire that assesses the impact of FM, in addition to the decrease in the patient’s functionality and quality of life, through questions about activities of daily living, pain, fatigue, memory, balance, sleep and psychological aspects. It was validated, adapted and translated into Portuguese in 2013 by Paiva [18].

Body mass index (BMI) is an index that assesses nutritional status, which expresses the relationship between body weight and height (kg/m2). A value below 18.5 indicates malnutrition. Other parameters are: 18.5 to 24.9 (normal weight), 25 to 29.9 (overweight), a value greater than or equal to 30 indicates obesity, with a value between 30 and 34.9: grade I obesity, between 35 and 39.9 grade II obesity and above or equal to 40 grade III obesity [19].

The Beck Depression Inventory is a self-reported scale composed of 21 items with a response from zero to three that assesses the degree of depression of the patient. According to the sum of the questions, it can be classified as follows: 0 to 9 (individual without depression), 10 to 18 (mild to moderate depression), 19 to 29 (moderate to severe depression) and 30 to 63 (severe depression). It was validated and adapted to the Portuguese language in 2012 by Gomes-Oliveira [20].

A Numeric Rating Scale of sleep quality (NRS) is an assessment of only one item (sleep quality) defined by choosing a number that best describes the quality of your sleep in the last 24 h. A score is assigned to the item, ranging from 0 to 10, where 0 is the best possible sleep and 10 the worst possible sleep.

### Evaluation of *IL8* gene expression

Blood collection was performed in both groups, by peripheral puncture of the brachial vein, with a syringe of 10 milliliters. The blood sample was stored in an EDTA (Ethylenediaminetetraacetic acid) tube. Total blood RNA extraction was performed using the QIAamp RNA Blood Mini Kit (Qiagen) kit and, afterward, all samples were quantified by the NanoDrop^®^ 2000 Spectrophotometer (Thermo Scientific, Wilmington, DE). The cDNA was obtained from the QuantiTect^®^ Reverse Transcription Kit (Qiagen) following the manufacturer’s specification. qPCR analysis was performed on StepOnePlus™ equipment (Applied Biosystems), using the GoTaq^®^ qPCR Master Mix kit (Promega). The cDNA samples were quantified and diluted to 100ng/µL. The b-actin gene was used as a reference gene, and, for the analysis of IL8 expression, specific primers were used at a concentration of 10pM (IDT). The relative expression of *IL8* was determined with the comparative cycle threshold (Cq) (2-^△△Cq^) method. The samples were evaluated in duplicate and those with a standard deviation of less than 0.5 from the Cqs of the duplicates were considered.

### Statistical analysis

The statistical analysis was performed using the GraphPad Prism Software and the data was correlated with previously obtained clinical information from the patients. In all groups, normality was verified using the Shapiro-Wilk test and the adequate test was performed according to the normality (Student’s T-test or Kruskall-Wallis test). To verify the association between quantitative variables, Pearson’s chi-square test was applied.The value of *p* < 0.05 was considered significant for the analysis.

## Results

**Fig. 1:**
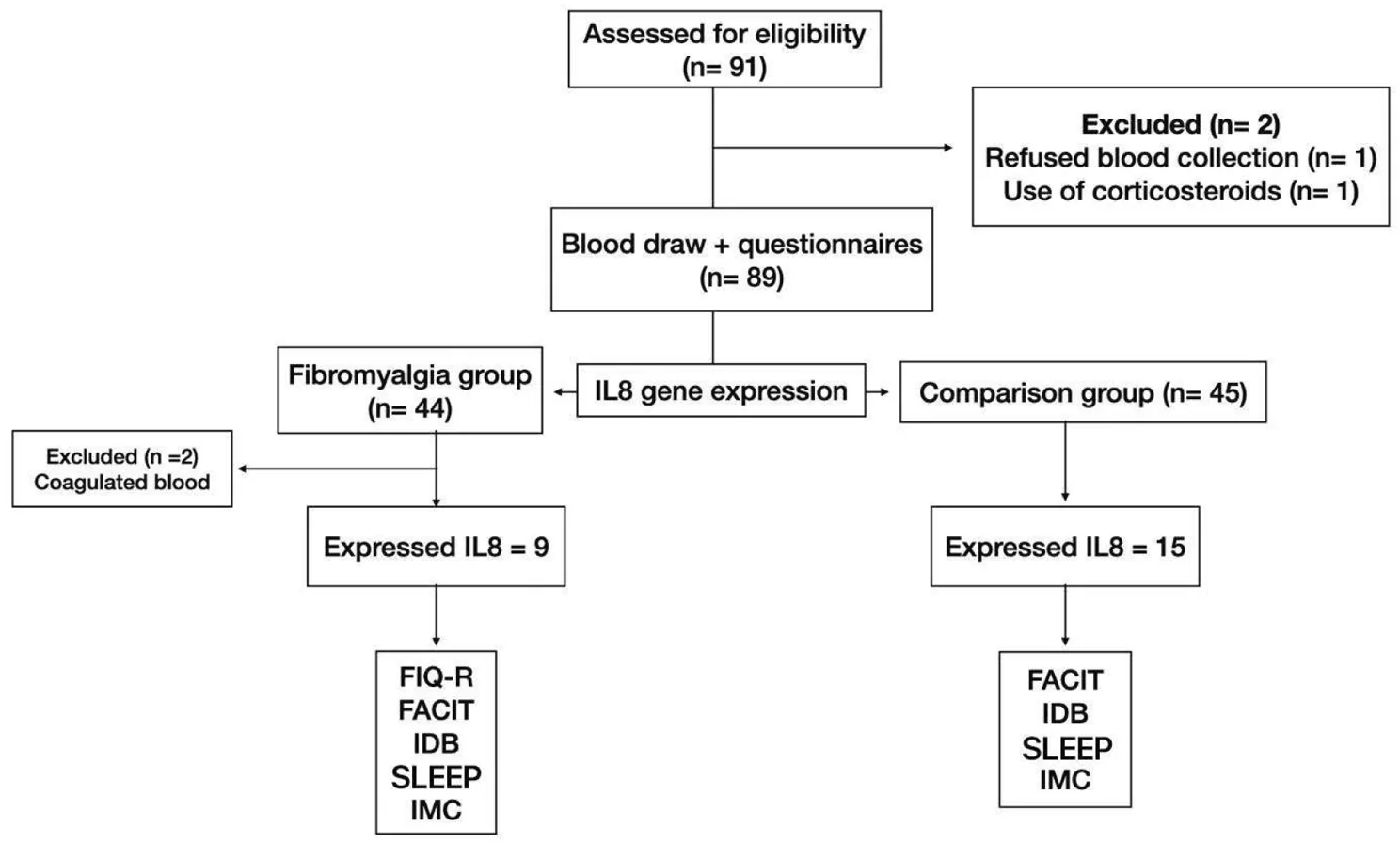
Study flowchart. FACIT: Functional Assessment of Chronic Illness Therapy; FIQ-R: Fibromyalgia Impact Questionnaire-Revised; BDI: Beck depression inventory; BMI: Body mass index

A total of 91 patients were considered for inclusion in the study, however 2 were excluded for refusing blood collection and another for corticoid use. It was included 89 participants, 44 patients from the FMG and 45 individuals from the CG, all women aged between 18 and 60 years. As described in the study flowchart, shown in Fig. 1, qPCR blood analysis was performed in only 42 from 44 participants in FMG because in 2 samples occurred coagulation of the blood in the tube.

The comparison of clinical and demographic characteristics of FMG and CG are present in Table 1. Regarding comorbidities, the FMG had a higher frequency of depression (34.1%), diabetes (25%) and hypertension (36.4%). Dyspareunia was present in 47.7% of FMG, the difference being statistically significant when compared to the CG, where it was only reported in one patient (*p* < 0.001). The frequency of obesity, assessed by BMI, was higher among patients with FM (52.5%), while in the CG the percentage of obesity was 29.5%. Regarding the practice of physical activity, in the FMG 31 (70.5%) patients did not do any physical activity versus 24 (43.6%) in the CG (*p* = 0.038). In the FACIT-F questionnaire, the mean score in the FMG was lower than that observed in the CG (*p* < 0.001). The average of NRS and BDI scales were significantly higher in the FMG (*p* < 0.001). A difference related to the frequency of depression considered moderate and severe in FMG (*p* < 0.001). Regarding FMG, the median time of diagnosis of FM was 10 years, mean time of pain was 13 years and median FIQ-R was 86.7. As for medications, the most frequently used was amitriptyline (24.7%), followed by gabapentin (19.1%), duloxetine (14.6%) and cyclobenzaprine (10.1%). No association was observed between the medians of the BDI, NRS, FACIT-F and FIQ-R scales with BMI stratified into eutrophic, overweight and obese in the FMG and of the BDI, NRS, FACIT-F scales in the CG.

**Table 1:** Comparison of clinical and demographic characteristics of fibromyalgia patients and comparison group

Characteristics	FMG (*n* = 44)	CG (*n* = 45)	*P*-value*
**Age (mean**,** SD)**	42.6 (7.2)	42.5 (7.0)	0.945
**Race (n**,** %)**			
White	11 (25.0%)	18 (40.0%)	**0.033**
Brown	31 (70.5%)	20 (44.4%)	
Black	2 (4.5%)	7 (15.6%)	
**Work (n**,** %)**			
Unemployed	25 (56.8%)	0 (0%)	**0.001**
Manual work	12 (27.3%)	4 (8.9%)	
Intellectual work	4 (9.1%)	40 (88.9%)	
Away on leave	3 (6.8%)	1 (2.2%)	
**Marital Status (n**,** %)**			
Married	23 (52.3%)	19 (42.2%)	0.157
Single	12 (27.3%)	21 (46.7%)	
Widow	2 (4.5%)	0 (0%)	
Divorced	7 (15.9%)	5 (11.1%)	
**Comorbidities (n**,** %)**			
Depression	15 (34.1%)	3 (6.7%)	**0.001**
Diabetes	11 (25.0%)	2 (4.4%)	**0.007**
Hypertension	16 (36.4%)	1 (2.2%)	**0.001**
Dyspareunia	21 (47.7%)	0 (0%)	**0.001**
Asthma	0 (0%)	3 (6.7%)	0.242
Dyslipidemia	3 (6.8%)	0 (0%)	0.117
Osteoporosis	1 (2.3%)	1 (2.2%)	0.999
Arthrosis	3 (6.8%)	0 (0%)	0.117
Smoking	1 (2.3%)	2 (4.4%)	0.999
Illicit drugs	0 (0%)	1 (2.2%)	0.999
**Boby mass index (n**,** %)**			
Normal	8 (18.2%)	20 (45.5%)	**0.018**
Overweight	13 (29.5%)	11 (25.5%)	
Obesity	23 (52.3%)	13 (29.5%)	
**Does physical activity**	0 (0%)	6 (13.3%)	**0.012**
Aerobic	10 (22.7%)	8(17.8%)	0.561
Resistance	2 (4.5%)	8 (17.8%)	0.090
Ambos	0 (0.0%)	6 (13.3%)	**0.026**
No activity	31 (70.5%)	24 (43.6%)	**0.038**
**FACIT-F (mean**,** SD)**	14.64 (7.28)	39.84 (9.23)	**< 0.001**
**NRS (mean**,** SD)**	8.73 (1.82)	2.56 (1.57)	**< 0.001**
**BDI (mean**,** SD)**	20.23 (10.13)	7.52 (6.07)	**< 0.001**
**Depression (n**,**%)**			
Without depression	0 (0%)	32 (72.7%)	**< 0.001**
Mild depression	5 (11.4%)	9 (20.4%)	
Moderate depression	20 (45.4%)	3 (6.8%)	
Severe depression	19 (43.2%)	0 (0%)	

FMG: Fibromyalgia group; CG: Comparison group; FACIT-F: Functional Assessment of Chronic Illness Therapy; NRS: Numeric Rating Scale of sleep quality; BDI: Beck depression inventory. SD: Standard Derivation

*CI: 95%

**Fig. 2:**
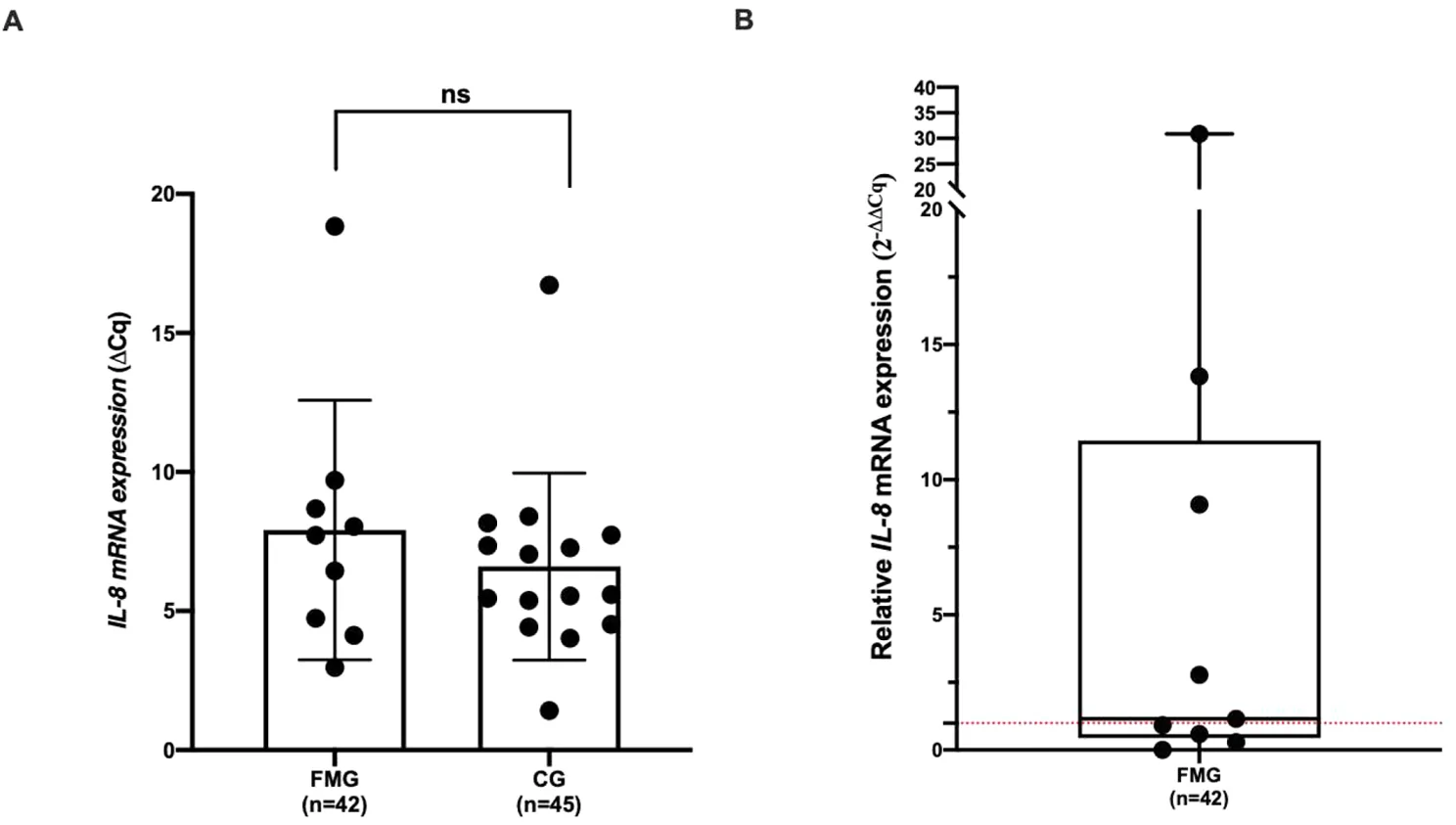
*IL8* mRNA expression in patients with fibromyalgia. **A*** IL8* mRNA expression in patients with fibromyalgia in relation to CG. There is no significant (ns) difference between the groups regarding the *IL8* expression. **B** Relative *IL8* mRNA expression in FMG. From 9 patients, 5 presented up regulated in relation to CG

In the evaluation of *IL8* gene expression, 87 of the 89 samples were evaluated, with two being excluded due to blood clotting. Among the 87 samples, 42 were from FMG and 45 from CG. *IL8* mRNA expression was detected in 15 individuals from the CG while only 9 patients from the FMG had the gene expression detected. There was no significant difference in △Cq between groups (Fig. 2). There was also no significant difference in the relative expression (2^−△△Cq^) between the groups, and 5 from 9 individual those present the *IL8* expression in FGM were up-regulated in relation to individuals from the CG.

**Fig. 3:**
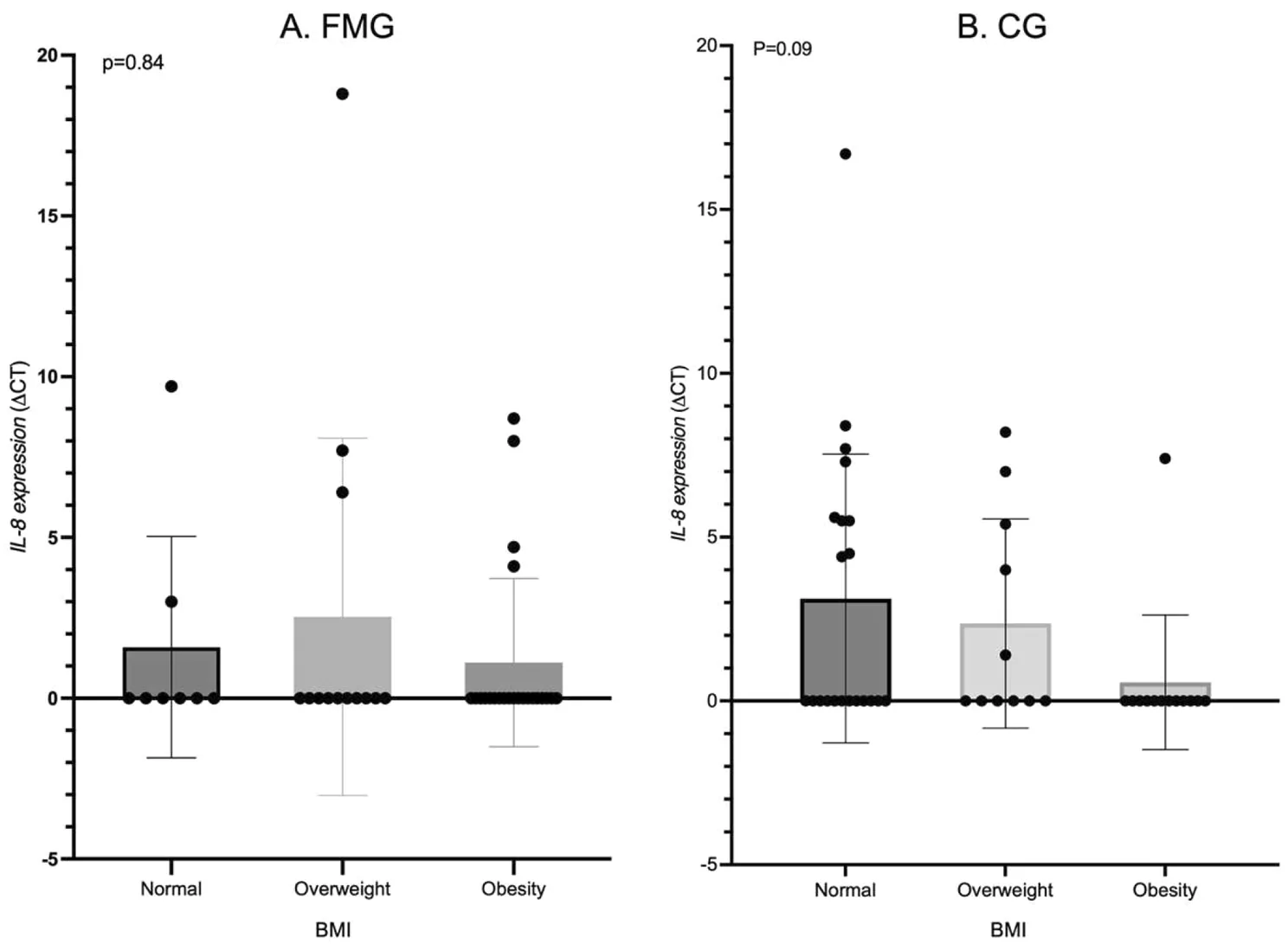
Association between IL8 gene expression (deltaCT) with body mass index in FMG (**A**) and CG (**B**). FMG: Fibromyalgia group; CG: Comparison group

No association was observed between *IL8* gene expression and BMI value (Fig. 3), NRS, FIQ-R and FACIT-F. When evaluating *IL8* and physical activity, it was observed that FMG patients who did not express IL8 were those who practiced less physical activity (*p* = 0.039).

**Fig. 4:**
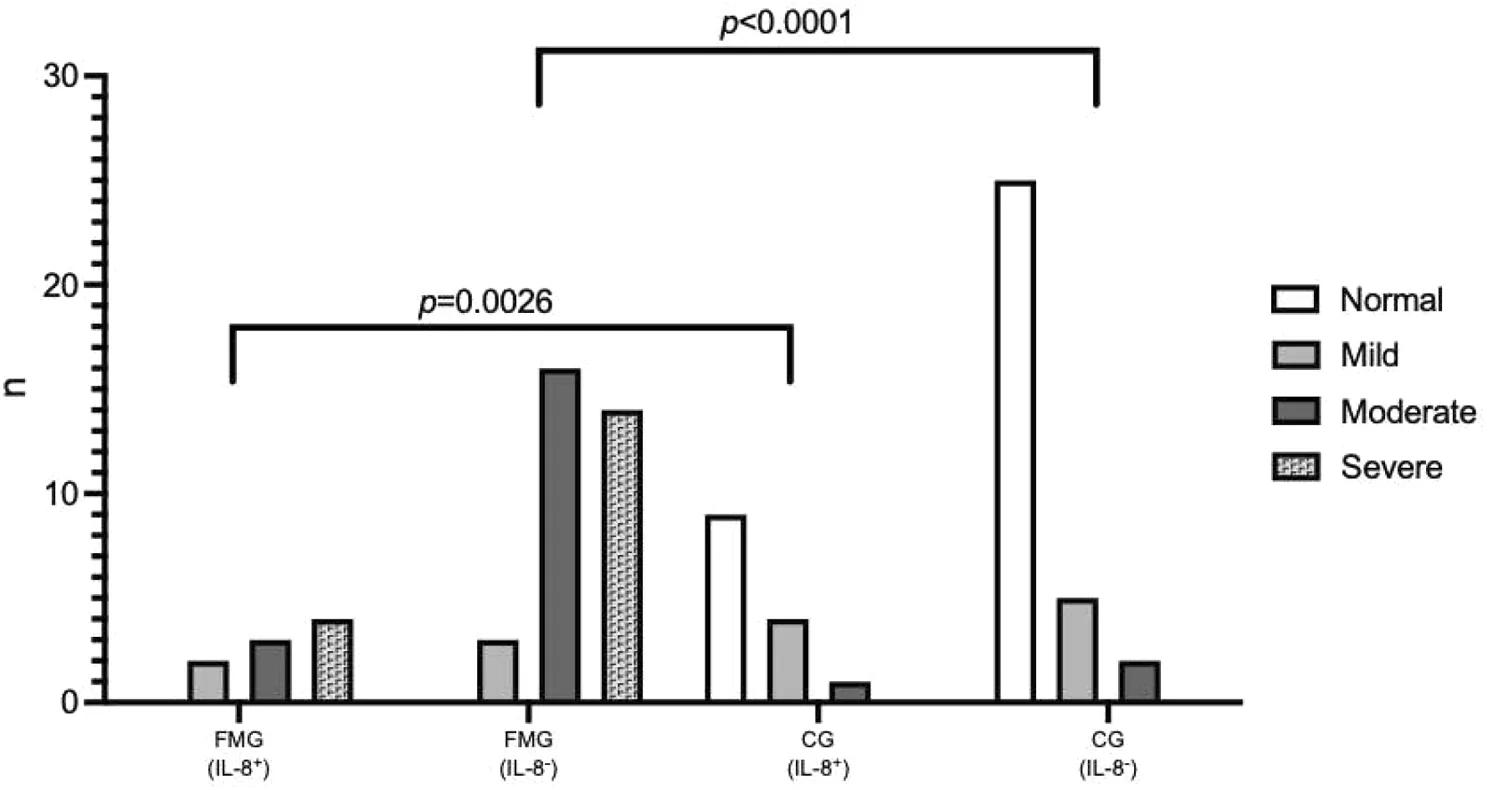
Association of IL8 gene expression (deltaCT) with Beck Depression inventory stratified into normal, mild, moderate and severe. FMG: Fibromyalgia group; CG: Comparison group

Regarding depression, it is observed that among the group with *IL8* expression (FMG and CG), worse depression rates were observed (*p* = 0.0026) as shown in Fig. 4.

Regarding the practice of physical activity, 70.5% of the FMG did not practice versus 46.6% on CG. When evaluating exercise with the scales, among patients with FM, there was an association between physical inactivity and worse depression rates (*p* = 0.019). There was an association in FMG between worse fatigue rates among sedentary individuals (FMG *p* = 0.004). Regarding the impact of FM, there was a higher median FIQ-R among cases that did not do physical activity (*p* = 0.016) as shown in Table 2.

**Table 2:** Association of physical activity with sleep scale, fatigue and depression in fibromyalgia and comparison group

Characteristics	Does physical activity	No physical activity	*P*-value*
**Fibromyalgia group**			
Sleep scale - Median (IQR)	9 (7.5- 9)	10 (8–10)	0.105
BDI - Median (IQR)	23 (14.5–29)	29.5 (25–36.5)	**0.017**
Classification - number (%)			
Mild depression	4 (33.3%)	1 (3.1%)	**0.019**
Moderate depression	5 (41.7%)	15 (46.9%)	
Severe depression	3 (25%)	16 (50%)	
FACIT-F – Median (IQR)	20.5 (13–25.5)	11 (7–16.5)	**0.004**
FIQ-R – Median (IQR)	79.3 (61.5–85.5)	89.2 (81.2–95.9)	**0.016**
**Comparison group**			
Sleep scale Median (IQR)	2 (1–3)	2 (1–5)	0.265
BDI – Median (IQR)	4 (3–6)	7 (4–10)	**0.028**
Without depression	14 (82.3%)	18 (66.7%)	0.665
Mild depression	2 (11.8%)	7 (25.9%)	
Moderate depression	1 (5.9%)	2 (7.4%)	
FACIT-F – Median (IQR)	44 (40–49)	39 (32–47)	0.052

IQR: Interquartile range; BDI: Beck depression inventory; FACIT-F: Functional Assessment of Chronic Illness Therapy; FIQ-R: Fibromyalgia Impact Questionnaire-Revised

*CI: 95%

## Discussion

Searching in the literature, so far, there is no research evaluating *IL8* gene expression in peripheral blood samples, only analysis in serum (plasma) and CSF dosage in patients with FM. Therefore, there is a difficulty in comparing our results with those in the literature. Previous studies indicate that IL8 concentration has an important clinical role in FM, mainly associated with pain and sleep disturbances [21, 22]. However, our study shows that there is a lower frequency in the expression of *IL8* in individuals with FM, and *IL8* expression was not associated with worsening of the disease, since there was no association with FIQ-R, sleep disturbance, BMI or fatigue.

The *IL8* expression occurred in a greater number of individuals without FM and *IL8* mRNA was mainly upregulated in individuals with FM, but in the present study, no difference was observed between *IL8* gene expression in patients with FM compared to healthy individuals. Also, patients with FM were mostly upregulated, although there is no cut off in the literature to *IL8* gene expression in patients with FM. This data indicated a greater stimulus to express the *IL8* gene in more than half patients with FM, but several post-transcriptional regulation mechanisms can influence the conversion of RNA to protein. Then, a better understanding of post-transcriptional regulation would be important to understand the role of up regulated mRNA *IL8* in individuals with FM in our results.

Furthermore, there was no difference in obese individuals, where we expected an increase in IL8 given the inflammatory state generated by obesity and other studies using various serum interleukins. We observed that regardless of trophism, there was no increase in IL8 expression, which may represent that obesity, depending on the degree, does not activate gene expression, but has the ability to generate interleukin in large amounts in peripheral blood. These results may show that there must be a different behavior between gene expression and serum level, also because there is more than one stimulus pathway for IL8 production.

When comparing our study with previous ones that analyze plasma levels of Interleukin 8 the results are conflicted. The first meta-analysis evaluating IL8 in 2011 showed an increase in IL8 serum levels in FM group [21]. Other studies also found IL8 increased in the cerebrospinal fluid [23] and peripheral blood [24–26] and. More recently, in 2021, a meta-analysis showed increased serum levels of IL8 in patients with FM compared to the group of healthy patients [15]. A Brazilian study in 2016 evaluating IL8 and other cytokines, showed no increase in serum IL8 and no association with disease severity, assessed by FIQ-R, or with depression [25]. Our results are concordant with this one, but we found an association between *IL8* gene expression and worse depression scores by the BDI. As in our results, a recent study also did not find a difference in IL8 concentration comparing patients with FM and healthy ones [26].

In addition to contradictory studies, even within the same research group, recent systematic reviews show very small studies (with less than 20 patients) and without rigorous exclusion criteria such as infection, making the serum assessment unreliable [11]. Another important factor that makes it difficult to compare with data from the present study is the way of assessing interleukin (its serum measurement) where there is known to be a lot of interference, large variations during the day, which may change with infection and use of medications.

Beyond Fibromyalgia, other chronic pain syndromes may be linked to IL8. A previous study analyzing the CSF of patients with chronic low back pain found elevated IL8 levels compared to pain-free individuals. Moreover, inhibiting IL8 with reparixin in rats led to a reduction in pain-related behaviors [12]. Similarly, patients with chronic pain after laminectomy exhibited higher IL-8 levels in CSF than controls [27]. Lastly, IL-8-reducing polymorphisms (IL-8 rs4073A > T) in patients with endometriosis have been associated with lower levels of chronic pelvic pain [28].

Evaluating gene expression with obesity, sleep disturbance, FIQ-R and physical activity, no association was seen in our study. There are studies in the literature that assess serum levels, with some studies demonstrating an association between better FIQ-R indices and lower plasma IL8 values [8]. There are no reports on gene expression level related to FIQ-R, FACIT or BMI.

In the present study, no difference was observed between trophism through BMI and IL8 expression. There is a report in the literature of the relationship between obesity and serum IL8 assessment, showing that patients with FM and high BMI (overweight and obesity) have higher levels of IL8 in the peripheral blood assessment [29], but there is no data linking obesity with gene expression.

In addition, other studies demonstrate high serum levels of IL8 in patients with obesity and a decrease in these indices of physical activity, as demonstrated in 2016, in a study comparing interleukin and degree of physical activity, observing that the more intense the exercise was seen a more significant decrease of IL8 in patients with FM and obesity [29]. In our study, it was seen that in the CG, patients who showed *IL8* expression did not practice physical activity. However, the practice of physical activity did not influence the frequency of *IL8* expression in the FM group.

Regarding the degree of fatigue, assessed by the FACIT, no association was seen between having more fatigue and presenting IL8 expression. There is no similar evaluation in the literature comparing gene expression with fatigue in the population with FM, but there are studies with fatigue and gene expression of *IL8*and *IL10* in other pathologies such as cancer, showing a significant association in the expression and worsening not only of fatigue, but also of pain and depression [30, 31]. Also, studies demonstrated an association between better FIQ-R indices and lower plasma IL8 values [30]. However, there is no data linking obesity and *IL8* gene expression, sleep disturbance, FIQ-R and physical activity in patients with FM.

Evaluating the BDI in our results, there is a difference in the number of individuals with moderate depression and *IL8* expression, showing that patients who expressed *IL8* were more frequently classified as having moderate depression. These results differ from another study that evaluated IL8 and IL6 in the population with FM, using serum dosage, where no association with mood disorder was seen [24].

In the analysis of BMI with the variables depression, fatigue and sleep, the present study did not show a significant difference, while other studies point to a correlation between depression and fatigue and obesity [32]. However, a more recent study with 239 patients with and without FM, using the same scale (Beck Depression Inventory), did not demonstrate a relationship between depression and obesity, as in the present study, observing, however, a relationship between the practice of physical activity and lower levels of depression [33].

A recent study in 2021 evaluated 2339 patients with FM separated into two groups: underweight and eutrophic versus overweight and obesity, concluding that in the overweight and obese group the FIQ-R values were much higher [19]. With regard to disease control, assessed by FIQ-R, it can be inferred that, if there were a larger sample of patients, a relationship with BMI would probably be found, as there was a tendency of association.

Regarding physical activity, an important component for the treatment of obesity and fibromyalgia, more than half of the FM group did not practice any physical activity. There was an association between practicing physical activity and being eutrophic, and also with worsening depression, confirmed by the BDI in addition to worse scores on FACIT and FIQ-R among those who did not do any physical exercise. This data corroborates the result of a randomized clinical trial where patients with FM were divided into groups with an aerobic physical activity program (walking), resisted (weight training) and in the group without follow-up physical activity, showing that those who exercised had a FIQ value -R significantly lower, and the walking group presented even lower values compared to resistance exercise [34]. In 2024, a randomized controlled trial showed higher levels of depression in women with FM compared with women without the disease and evaluated physical activity in the FM group, where after 4 weeks of resistance training, there was a difference in depressive symptoms [35]. In our study, we had only one patient who did resistance exercise, and we were unable to compare whether there was a difference between the modalities. In our study, we had only one patient who did resistance exercise, and we were unable to compare whether there was a difference between the modalities. An umbrella review, published in 2020, which analyzed 37 systematic reviews, showed that aerobic exercise and muscle strength training are the most used non-pharmacological treatments in FM, with strong evidence that adapted physical activity reduces pain perception and improves quality of life of patients with FM [36].

A study involving healthy volunteers who engaged in intense aerobic activity demonstrated a 68% rise in IL-8 levels post-exercise [37], indicating that exercise can trigger an acute inflammatory response, reflected by elevated IL-8 levels. However, chronic regular exercise appears to exert antiinflammatory effects. In individuals with chronic obstructive pulmonary disease, an eight-week physical training program led to a significant reduction in IL-8 levels in CD4 + T lymphocytes [38]. Regular exercises in patients with rheumatoid arthritis led to lower inflammatory gene expression, including IL8 [39]. Therefore, while acute physical activity appears to elevate IL-8 expression, consistent regular exercise seems to reduce it, potentially helping to alleviate chronic pain.

Some limitations should be considered in this study, such as the small sample of patients and the possibility of socioeconomic differences between the FMG and CG groups. This difference may reflect nutritional aspects, stress level, access to health and interfere with the *IL8* value. In addition to the composition of the sample, it should be noted that the sleep scale is a question that refers to the last day of sleep, which masks the overall sleep assessment because it considers only one day and is a self-report, which may have confounding factors for this note. Some studies use devices to assess sleep quality more objectively. Another limitation was not having evaluated the serum value of IL8 to understand how to improve if there is variation in plasma level between the groups, and to compare with data in the literature. Another important factor that makes it difficult to compare with data from the present study is the way of assessing interleukin (its serum measurement) where there is known to be a lot of interference, large variations during the day, which may change with infection and use of medications.

In conclusion, there is no difference in frequency of *IL8* gene expression when comparing individuals with FM and healthy. *IL8* gene expression was not associated with sleep, fatigue, obesity and FIQ-R among patients with FM, but there was an association between expressing *IL8* and having moderate depression. Patients with FM have a higher frequency of obesity, but the BMI value was not associated with worse sleep rate, fatigue, depression, FIQ-R and *IL8* expression.

## Data Availability

No datasets were generated or analysed during the current study.

## Abbreviations

BDI: Beck depression inventory
BMI: Body mass index
CG: Comparison group
CNS: Central nervous system
CRP: C reactive protein
CT: Threshold cycle
EGS: Symptom Severity Scale
FACIT: Functional Assessment of Chronic Illness Therapy
FIQ-R: Fibromyalgia impact questionnaire-revised
FM: Fibromyalgia
GDI: Generalized Pain Index
IL8: Interleukin 8
NRS: Numeric rating scale for sleep
TNF-α: Tumor necrosis factor alpha
WHO: World health organization
